# Association of Perceived Stress Levels With Long-term Mortality in Patients With Peripheral Artery Disease

**DOI:** 10.1001/jamanetworkopen.2020.8741

**Published:** 2020-06-23

**Authors:** Ali O. Malik, Poghni Peri-Okonny, Kensey Gosch, Merrill Thomas, Carlos Mena, William R. Hiatt, Philip G. Jones, Jeremy B. Provance, Clementine Labrosciano, Qurat-ul-ain Jelani, John A. Spertus, Kim G. Smolderen

**Affiliations:** 1 Department of Cardiovascular Medicine, University of Missouri, Kansas City, Kansas City; 2 Department of Cardiovascular Medicine, Saint Luke’s Mid America Heart Institute, Kansas City, Missouri; 3 Vascular Outcomes Program, Department of Internal Medicine, Yale University, New Haven, Connecticut; 4 Department of Medicine, University of Colorado, Aurora; 5 Department of Medicine, University of Adelaide, Adelaide, South Australia, Australia; 6 Department of Internal Medicine, Yale University, New Haven, Connecticut

## Abstract

**Importance:**

Identifying modifiable risk factors, such as stress, that could inform the design of peripheral artery disease (PAD) management strategies is critical for reducing the risk of mortality. Few studies have examined the association of self-perceived stress with outcomes in patients with PAD.

**Objective:**

To examine the association of high levels of self-perceived stress with mortality in patients with PAD.

**Design, Setting, and Participants:**

This cohort study analyzed data from the registry of the Patient-Centered Outcomes Related to Treatment Practices in Peripheral Arterial Disease: Investigating Trajectories (PORTRAIT) study, a multicenter study that enrolled patients with new or worsening symptoms of PAD who presented to 16 subspecialty clinics across the US, the Netherlands, and Australia from June 2, 2011, to December 3, 2015. However, the present study included only patients in the US sites because assessments of mortality for patients in the Netherlands and Australia were not available. Data analysis was conducted from July 2019 to March 2020.

**Exposure:**

Self-perceived stress was quantified using the 4-item Perceived Stress Scale (PSS-4), with a score range of 0 to 16. A score of 6 or higher indicated high stress in this cohort. Missing scores were imputed using multiple imputation by chained equations with predictive mean matching. Stress was assessed at baseline and at 3-, 6-, and 12-month follow-up. Patients who reported high levels of stress at 2 or more follow-up assessments were categorized as having chronic stress.

**Main Outcomes and Measures:**

All-cause mortality was the primary study outcome. Such data for the subsequent 4 years after the 12-month follow-up were obtained from the National Death Index.

**Results:**

The final cohort included 765 patients, with a mean (SD) age of 68.4 (9.7) years. Of these patients, 57.8% were men and 71.6% were white individuals. High stress levels were reported in 65% of patients at baseline and in 20% at the 12-month follow-up. In an adjusted Cox proportional hazards regression model accounting for demographics, comorbidities, disease severity, treatment type, and socioeconomic status, exposure to chronic stress during the 12 months of follow-up was independently associated with increased risk of all-cause mortality in the subsequent 4 years (hazard ratio, 2.12; 95% CI, 1.14-3.94; *P* = .02).

**Conclusions and Relevance:**

In thie cohort study of patients with PAD, higher stress levels in the year after diagnosis appeared to be associated with greater long-term mortality risk, even after adjustment for confounding factors. These findings suggest that, given that stress is a modifiable risk factor for which evidence-based management strategies exist, a holistic approach that includes assessment of chronic stress has the potential to improve survival in patients with PAD.

## Key Points

**Question:**

Is there an association between chronic stress and mortality risk in patients with peripheral artery disease?

**Findings:**

In this cohort study of 765 patients with new symptoms of peripheral artery disease, higher stress levels in the year after diagnosis were independently associated with higher risk of mortality in the subsequent 4 years.

**Meaning:**

The findings of this study suggest that because stress is a modifiable risk factor for which evidence-based management is available, holistic interventions should be tested to mitigate the implications of stress for outcomes in patients with peripheral artery disease.

## Teaser

This cohort study compares baseline characteristics and mortality risk in patients with peripheral artery disease who reported high levels of stress vs those who did not report such levels of stress.

## Introduction

Lower-extremity peripheral artery disease (PAD) is prevalent worldwide, with more than 200 million people estimated to have the disease in 2010.^1^ In the US, the age-standardized prevalence of PAD has been estimated to be approximately 12%.^2^ Patients with PAD are at a higher risk of premature death, and the 5-year mortality rate is nearly 25%.^3^ Incidence of disability associated with PAD is also high, adding directly and indirectly to the global economic burden of this disease.^4,5^ Given that a key goal of therapy in patients with PAD is to decrease the risk of premature death, identifying the various factors associated with mortality risk is critical for designing novel interventions that improve outcomes.

Stress is one such potential factor. The stress construct has been defined as a response, stimulus, and transaction.^6^ Stress occurs when demands exceed one's personal resources to cope with these challenges.^7,8^ High levels of perceived stress are a potent risk factor for the development and progression of coronary artery disease and cardiovascular death.^9,10^ High levels of perceived stress at the time of myocardial infarction have been associated with a 40% higher risk of dying in the 2 years after the event.^11^ Chronic exposure to stress may further complicate the management of and experiences with a devastating chronic disease, such as PAD.

A previous study reported that almost one-third of patients presenting with symptomatic PAD experience higher levels of stress than experienced in the overall population.^12^ Despite the prevalence of stress, the association of stress with long-term mortality risk in patients with PAD has not been explored. To address this gap in knowledge, we examined the association between exposure to perceived stress in the year after the initial PAD evaluation and mortality in the subsequent 4 years.

## Methods

### Study Population

This cohort study analyzed data from the registry of the Patient-Centered Outcomes Related to Treatment Practices in Peripheral Arterial Disease: Investigating Trajectories (PORTRAIT) study, which enrolled patients (n = 1275) with new or worsening symptoms of PAD who presented to 16 subspecialty clinics across the US, the Netherlands, and Australia from June 2, 2011, to December 3, 2015. Details of the PORTRAIT study have been described previously.^13^ Briefly, patients presenting to a specialty clinic with new or worsening symptoms of PAD and an ankle-brachial index (ABI) of 0.90 or less or a substantial decrease in postexercise ankle pressure (≥20 mm Hg) were enrolled. Patients with a noncompressible ABI of 1.30 or greater, critical limb ischemia, and lower-limb revascularization in the 12 months before a PAD visit and those who were incarcerated, hard of hearing, or unable to provide informed consent were excluded. The study protocol of the PORTRAIT study was approved by the institutional review boards of all 16 participating sites; this approval extends to the present study. All study participants provided either written or verbal (by telephone) informed consent. The present study followed the Strengthening the Reporting of Observational Studies in Epidemiology (STROBE) reporting guideline.^14^ Data analysis was conducted from July 2019 to March 2020.

**Figure 1.**
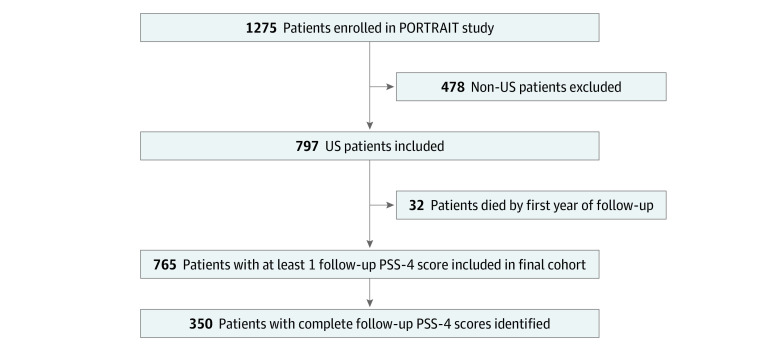
STROBE Diagram of Analytical Cohort PORTRAIT indicates Patient-Centered Outcomes Related to Treatment Practices in Peripheral Arterial Disease: Investigating Trajectories; PSS-4, 4-item Perceived Stress Scale.

Because accurate assessments of mortality for patients in the Netherlands and Australia were not available, only patients in the US clinics (n = 797) were included. After excluding 32 patients who had died in the first 12 months of follow-up, the final analytical cohort consisted of 765 patients. A STROBE diagram is presented in Figure 1.

### Definition of Chronic Stress at Follow-up

In the PORTRAIT study, each patient’s perception of stress and ability to cope with it were quantified with the 4-item Perceived Stress Scale (PSS-4). The PSS-4 is a reliable and valid tool (Cronbach α = 0.67-0.79) for self-evaluation of control and confidence in handling stressful situations in the past month.^15^ The cohort had Cronbach α = 0.66. The PSS-4 consists of 4 items scored on a 5-point Likert scale, with reverse coding for 2 items.^16^ Scores range from 0 to 16, with higher scores indicating higher levels of stress. No cutoff scores were established; instead, patient scores were compared with normative values from the general population. Warttig et al^17^ found that the normative score of an English population was 6. In a US cohort of patients with cardiovascular disease, a score of 6 was used to depict patients with high levels of perceived stress and was independently associated with adverse outcomes.^11^ In the present cohort, the distribution of baseline PSS-4 scores in the 25th percentile was 1, in the 50th percentile was 3, and in the 75th percentile was 7. Hence, given the previous work on the association of stress with cardiovascular disease outcomes and the distribution of PSS-4 scores in the PORTRAIT study, a PSS-4 score of 6 was used to categorize patients with high levels of self-perceived stress. Scores were obtained at baseline and at 3-, 6-, and 12-month follow-up. Patients were categorized as having chronic stress if they reported high levels of stress at 2 or more follow-up assessments.

### Primary Outcome

To obtain 4 years of vital status information for patients who were alive 12 months after their enrollment, we searched the National Death Index.^18^ The National Death Index provides reliable and accurate vital status data of US patients by identifying patient name, date of birth, and Social Security number.^19^ From this information, we established all-cause mortality as the primary study outcome. Although using the National Death Index to identify cause-specific mortality presents risk of misclassification, it is reliable for ascertaining all-cause mortality.^20^

### Other Variables

The PORTRAIT study obtained data on patient demographics, health status, psychosocial characteristics, and socioeconomic status by interview at the initial visit. Patients’ symptoms, medical history, comorbidities, and PAD diagnosis were abstracted from medical records. Socioeconomic status was assessed using patient responses to questions regarding their highest level of education (high school, college, or postgraduate), avoidance of care because of costs (yes or no), and funds left at the end of the month (some, just enough, or not enough).^21^ Severity of PAD was assessed using ABI at baseline. Treatment strategy at 3 months after a PAD examination was categorized as either invasive (including percutaneous or surgical interventions) or noninvasive (medical therapy only). Depressive symptoms at baseline were assessed with the 8-item Patient Health Questionnaire (PHQ-8) depression scale, a screening instrument for major depression.^22^ The PHQ-8 scores range from 0 to 24, with higher scores indicating greater severity of depressive symptoms. A score of 10 or higher implies clinically relevant symptoms that warrant further assessment.^22^

### Missing Data

In the final cohort of 765 patients, 217 patients had 1 missing follow-up PSS-4 score, 339 had 2 missing scores, and 76 patients had 3 missing scores. Most of these data were missing because the PSS-4 items from the 3- and 6-month interview forms were removed to reduce interview burden. Hence, the missingness in the follow-up data was primarily the result of an administrative decision and not likely caused by any patient factors.

To maximize the utility of the available follow-up data, we used multiple imputation by chained equations with predictive mean matching to impute missing PSS-4 scores.^23^ The imputation model included several baseline patient variables that were identified a priori on the basis of previous research and clinical judgment (eTable 1 in the Supplement). These variables included demographics (age, sex, and race/ethnicity), comorbidities (myocardial infarction, congestive heart failure, diabetes, hypertension, smoking, body mass index, and PHQ-8 score), socioeconomic indicators (end-of-month funds, avoidance of care because of cost, and educational level), and social support (ENRICHD Social Support Instrument^24^). In addition, the imputation model included the available PSS-4 scores at all time points and mortality data during follow-up. A total of 100 randomly imputed data sets were generated, and the outcome of chronic stress (defined as ≥2 follow-up PSS-4 scores indicating high levels of stress) was identified on each data set using observed and imputed scores. All analyses were performed by analyzing each of the 100 data sets separately and then pooling the results. This approach accounted for bias and uncertainty owing to missingness.

To ensure the validity of the imputed data set, we compared the proportion of patients with chronic stress and baseline characteristics between patients with complete follow-up PSS-4 scores (n = 350) (eTable 2 in the Supplement) and patients with the imputed data. We also examined age-adjusted hazard ratio (HR) of mortality during follow-up in the 2 data sets.

Given the nature of the imputation, the assignment of patients to chronic stress groups varied across imputed data sets. Hence, in the Results section, instead of including the numbers with percentages, we reported only percentages, which represented the mean of the pooled results across the imputations.

### Statistical Analysis

Using standardized differences, we compared baseline patient characteristics between patients who did and patients who did not have chronic stress. The standardized difference, expressed as a percent, is the mean difference between groups divided by a pooled estimate of the SD. Unlike *P* values, standardized differences are not altered by sample size. Values greater than 10% were considered to suggest nontrivial differences between groups.

To examine the association between 12-month chronic stress and 4-year all-cause mortality after the initial 12-month follow-up period, we performed a landmark analysis with time 0 defined at 12 months. We constructed age-adjusted survival curves from a Cox proportional hazards regression model to compare the risk of all-cause mortality in the next 4 years between patients with and patients without chronic stress during follow-up.

To examine the independent association of chronic stress over the 12-month follow-up with all-cause mortality over the subsequent 4 years, we estimated HRs using a site-stratified Cox proportional hazards regression model adjusted for patient-level factors associated with mortality in patients with PAD. We identified these factors a priori according to previous research.^25^ These factors included demographics (age, sex, and race/ethnicity), comorbidities (diabetes, hypertension, history of myocardial infarction, congestive heart failure, and smoking), disease specifics (ABI and treatment type), medication use (aspirin, statin, and clopidogrel bisulfate), and socioeconomic status (educational level, avoidance of care because of cost, and end-of-month funds). Depression has been shown to be associated with perceived stress in patients with cardiovascular disease.^26^

To examine whether chronic stress was associated with mortality, independent of baseline depressive symptoms, we performed a secondary analysis that adjusted for baseline PHQ-8 score in the Cox proportional hazards regression model. To understand whether baseline stress moderated the association between chronic stress and mortality, we assessed the interaction between baseline stress and chronic stress toward the outcome of mortality.

All of these models included restricted cubic splines for estimating the associations between continuous variables to accommodate nonlinear associations. When no substantial evidence of nonlinearity was found, associations were reestimated using linear effects to simplify interpretation. The proportional hazards assumption was met. All statistical analyses were performed with SAS, version 9.4 (SAS Institute Inc). All statistical tests were 2-tailed, and statistical significance was determined using α = .05.

## Results

The final analytical cohort included 765 patients. In the final pooled analysis from 100 imputed data sets, the mean (SD) age was 68.4 (9.7) years; 57.8% were men and 71.6% were white individuals. High stress levels were reported in 65% of patients at baseline and in 20% at the 12-month follow-up. Table 1 shows the comparison of baseline characteristics between patients with and patients without chronic stress at follow-up. As previously mentioned, because of the nature of the imputation process, only percentages (not numbers) were reported, which represented the mean of the pooled results.

**Table 1.** Baseline Characteristics of Patients at Follow-up^a^

Variable	Percentage			
	Total	With chronic stress (17.6%)	Without chronic stress (82.4%)	Standardized difference
Demographics				
Age, mean (SD), y	68.4 (9.7)	63.4 (10.5)	69.5 (9.1)	62.2
Female sex	42.2	56.6	39.1	35.5
Male sex	57.8	43.4	60.9	35.5
White race/ethnicity	71.6	59.7	74.2	31.3
Comorbidities				
Current smoker	30.6	44.1	27.8	34.5
Diabetes	38.7	42.6	37.9	9.7
Hypertension	88.8	87.2	89.1	5.8
CHF	13.5	16.1	12.9	9.1
CKD	14.5	16.3	14.1	6.1
Cancer	9.4	10.3	9.2	3.5
Sleep apnea	11.0	10.5	11.1	2.1
Prior MI	22.0	25.6	21.2	10.4
BMI, mean (SD)	29.5 (6.3)	29.6 (6.6)	29.5 (6.2)	1.2
Prior stroke/TIA	11.1	14.6	10.4	12.8
Baseline PHQ-8 score, mean (SD)	4.8 (5.3)	10.2 (6.4)	3.6 (4.1)	123.4
PAD severity and treatment				
Invasive treatment	28.6	22.0	30.0	18.3
ABI, mean (SD)	0.68 (0.19)	0.68 (0.19)	0.68 (0.19)	1.2
Socioeconomic factors				
>High school degree	85.6	73.0	88.3	39.6
Not enough month-end funds	11.9	25.0	9.1	43.3
Avoidance of care because of cost	16.6	31.5	13.4	44.3
Medications at baseline				
Aspirin	83.1	78.1	84.2	15.7
Clopidogrel bisulfate	36.1	41.3	35.0	13.0
Statin	80.9	77.2	81.7	11.0

Abbreviations: ABI, ankle-brachial index; BMI, body mass index (calculated as weight in kilograms divided by height in meters squared); CHF, congestive heart failure; CKD, chronic kidney disease; MI, myocardial infarction; PAD, peripheral artery disease; PHQ-8, 8-item Patient Health Questionnaire depression scale; TIA, transient ischemic attack.

^a^ Because of the nature of the imputation, the assignment of patients to chronic stress groups varied across imputed data sets. Hence, in the Results section, instead of including the numbers with percentages, we reported only percentages, which represented the mean of the pooled results across the imputations.

In the pooled analysis, 17.6% of the patients had chronic stress during the 12-month follow-up (≥2 follow-up assessments with high levels of stress). Patients with chronic stress, compared with those without chronic stress, were younger (mean [SD] age, 63.4 [10.5] years vs 69.5 [9.1] years), were more likely to be female (56.6% vs 39.1%), were less likely to be white individuals (59.7% vs 74.2%), and had a higher burden of smoking (44.1% vs 27.8%) and history of myocardial infarction (25.6% vs 21.2%). Moreover, patients with chronic stress vs those without chronic stress had higher baseline mean (SD) PHQ-8 scores (10.2 [6.4] vs 3.6 [4.1]), and a higher proportion of these patients reported avoiding care because of cost (31.5% vs 13.4%).

**Figure 2.**
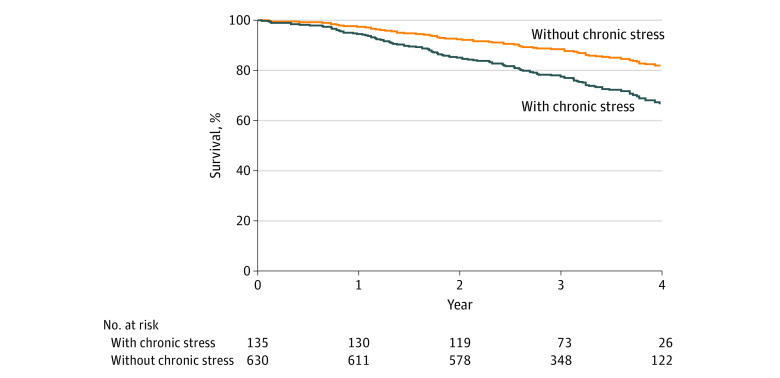
Association of Chronic Self-Perceived Stress and Mortality Age-adjusted survival curves in patients with chronic stress vs those without (*P* = .008).

Figure 2 shows age-adjusted survival curves for patients with or without chronic stress. Table 2 shows the association of chronic stress over a 12-month follow-up period with mortality over the subsequent 4 years, adjusted for confounding factors. The HR for chronic stress was 2.12 (95% CI, 1.14-3.94; *P* = .02). With additional adjustment for baseline PHQ-8 scores, no appreciable change in the effect size was found (HR, 2.06; 95% CI, 1.02-4.18; *P* = .045). The interaction between baseline stress and chronic stress at follow-up, toward the outcome of mortality, was not significant (HR for patients with baseline stress, 1.43 [95% CI, 0.61-3.36]; HR for patients without baseline stress, 2.94 [95% CI, 1.31-6.63]; *P* for interaction = 0.18).

**Table 2.** Association of Chronic Stress With Mortality, Adjusted for Confounding Factors

Variable	Hazard ratio (95% CI)	*P* value
Chronic stress	2.12 (1.14-3.94)	.02
Demographics		
Age	1.07 (1.05-1.10)	<.001
Female sex	0.82 (0.55-1.21)	.31
White race/ethnicity	1.09 (0.66-1.79)	.74
Comorbidities		
CHF	2.39 (1.57-3.66)	<.001
Prior MI	1.77 (1.15-2.71)	.009
Smoking	1.47 (0.93-2.33)	.10
Diabetes	1.41 (0.96-2.09)	.08
Hypertension	1.18 (0.60-2.33)	.63
Socioeconomic		
Not enough month-end funds	1.13 (0.60-2.15)	.70
Avoidance of care because of cost	0.78 (0.45-1.36)	.38
High school degree	0.77 (0.46-1.30)	.33
PAD severity and treatment		
Aspirin use	1.09 (0.65-1.83)	.74
Statin use	0.99 (0.61-1.60)	.96
Clopidogrel use	1.00 (0.68-1.48)	.99
Invasive treatment	0.81 (0.51-1.30)	.39
ABI	0.35 (0.13-0.96)	.04

Abbreviations: ABI, ankle-brachial index; CHF, congestive heart failure; MI, myocardial infarction; PAD, peripheral artery disease.

Baseline characteristics of the patient cohort with complete follow-up PSS-4 scores are described in eTable 2 in the Supplement. The patient demographics, comorbidities, and socioeconomic factors in this cohort were similar to those in the imputed data set. The age-adjusted HR for the association between chronic stress and mortality in this cohort was 2.61 (95% CI, 1.36-5.00).

## Discussion

With a growing population of patients presenting with PAD, examining the factors associated with worse outcomes is a critical strategy for identifying potential novel therapeutic strategies to further improve care. Using real-world, contemporary data of patients with new or worsening PAD symptoms, we found that 1 in 6 patients with PAD continued to have high levels of stress during the 12 months of follow-up. Higher levels of exposure to chronic stress were significantly associated with an increased risk of mortality, independent of patients’ baseline ABI, major comorbidities, treatment type, and socioeconomic status. We believe that this study has demonstrated the prognostic role of chronic stress in PAD and sets the stage for further exploration of holistic stress reduction strategies to improve outcomes for patients with PAD.

Previous studies have shown that perceived stress and a distressed personality are commonly present in patients with PAD and are associated with worse health status and quality of life.^27,28^ One study has found that mental health concerns, particularly stress, are prevalent in patients with PAD, especially at first presentation with new or worsening symptoms.^12^ We believe that the present study extends these observations by demonstrating that high levels of stress over the 12 months of follow-up were associated with increased mortality risk in these patients. Psychosocial factors such as stress have also been found to increase the risk of cardiovascular events and worsen the prognosis in other cardiovascular patient populations.^8,29^ Although in this study the prevalence of stress appeared to be disproportionately higher in patients with PAD, to our knowledge, no previous study has examined the association between stress and mortality in this population, underscoring the clinical significance of chronic stress.

Chronic perceived stress has been associated with adverse health behaviors such as physical inactivity,^30^ lifestyle habits–related obesity,^31^ and smoking.^32^ In younger women, disproportionate exposure to societal psychosocial stressors has been associated with adverse outcomes after myocardial infarction.^33^ In addition, various adverse physiological processes are triggered by exposure to chronic stress, including cardiovascular reactivity that has been associated with endothelial cell damage, inflammation, platelet reactivity, and the formation of atherosclerotic lesions.^34,35^ Moreover, slower recovery from sympathetic activity has been shown to be associated with stress, decreased heart rate variability, and increased blood pressure.^36^ These mechanisms warrant further study to explain the higher mortality risk that we observed in the PAD cohort.

Chronic stress can manifest in the context of other psychiatric conditions, such as depression, or even without a clinically diagnosed mental condition. Various idiosyncratic triggers can overwhelm or stress individuals, such as marital and financial strain^37,38^ and job insecurity.^39^ Regardless of its trigger or root cause, chronic stress is associated with increased cardiovascular risk.

However, stress is also known as a modifiable risk factor for which evidence-based management strategies exist.^40^ Coping skills and stress reduction have been associated with improved quality of life in patients with coronary artery disease.^41^ Stress management learned through cognitive behavioral therapy programs^42^ and transcendental meditation,^43^ in addition to standard care, have also been associated with reduced risk of recurrent cardiovascular events in patients with coronary artery disease and longer life expectancy in women after acute myocardial infarction.^44^ Given the associations found in this study and that stress has been largely ignored as a risk factor in PAD, future research is needed to test the implications of stress management strategies for cardiovascular outcomes in patients with PAD.

### Limitations

This study has some limitations. First, we had a high proportion of patients who had missing follow-up PSS-4 scores. However, as noted, most of this missingness was attributable to administrative changes to the 3- and 6-month interview forms. Hence, it is unlikely that the missingness introduced bias. Furthermore, the use of multiple imputation allowed us to account for potential biases associated with other observed factors, as the imputation model included many patient-specific factors that have been associated with chronic stress and mortality outcome. Second, most of the PORTRAIT study sites that recruited patients may not necessarily be representative of other PAD clinics that were not represented in the present study. Third, this analysis was restricted to US patients only, and these findings need to be replicated outside the US context. However, no evidence exists that suggests the association between chronic stress and clinical outcomes in cardiovascular disease would be different across geographical contexts.^45^

## Conclusions

In this cohort study involving a large, multicenter, contemporary data set of patients presenting with new or worsening symptoms of PAD, we found an independent association between higher levels of perceived stress in the year after a PAD diagnosis and increased mortality risk in the subsequent 4 years. These findings highlight the potential advantage of a holistic management approach that includes assessment of chronic stress. Such a strategy to improve the outcomes of patients with PAD should be tested in future research.

## Notes

Supplement. **eTable 1.** Patient Factors in the Multiple Imputation Model **eTable 2.** Baseline Patient Characteristics in Patient Cohort With Complete (At Least Two) Follow-up PSS-4 Scores
