# Supplementary material for: Association of Perceived Stress Levels With Long-term Mortality in Patients With Peripheral Artery Disease
Source: JAMA Netw Open. 2020 Jun 23;3(6):e208741. doi: 10.1001/jamanetworkopen.2020.8741 (PMC7312389; doi:10.1001/jamanetworkopen.2020.8741)
Supplement: Supplement. — eTable 1. Patient Factors in the Multiple Imputation Model eTable 2. Baseline Patient Characteristics in Patient Cohort With Complete (At Least Two) Follow-up PSS-4 Scores [file jamanetwopen-3-e208741-s001.pdf]

Supplementary Online Content

Malik AO, Peri-Okonny P, Gosch K, et al. Association of perceived stress levels with long-term mortality in patients with peripheral artery disease. *JAMA Netw Open*. 2020;3(6):e208741. doi:10.1001/jamanetworkopen.2020.8741

eTable 1. Patient Factors in the Multiple Imputation Model

eTable 2. Baseline Patient Characteristics in Patient Cohort With Complete (At Least Two) Follow-up PSS-4 Scores

This supplementary material has been provided by the authors to give readers additional information about their work.

©2020 Malik AO et al. *JAMA Network Open*

eTable 1. Patient Factors in the Multiple Imputation Model

Demographics
Age, Sex, Race
Comorbidities
Diabetes, Hypertension, Current Smoking Status, Prior Myocardial Infarction, Congestive Heart Failure, Body Mass Index, PHQ-8 score
Socioeconomic Indicators
Highschool Education, Not enough finances at months end, Avoiding care due to costs, Enriched Social Support Index
Other Patient Factors
Available PSS-4 score at all time points, Ankle Brachial Index, Invasive treatment strategy, Mortality, Medications at baseline
PHQ-8=8-point Patient Health Questionnaire, PSS-4= 4-point Perceived Stress Scale

©2020 Malik AO et al. *JAMA Network Open*

eTable 2. Baseline Patient Characteristics in Patient Cohort With Complete (At Least Two) Follow-up PSS-4 Scores

	**Total n=350**	**Chronic stress n=46**	**No chronic stress n=304**	**Standardized Difference**
Demographics				
Age (Mean ± SD)	68.5 ± 9.7	63.2 ± 12.7	69.3 ± 9.0	55.4%
Female Sex	134 (38.3%)	24 (52.2%)	110 (36.2%)	32.6%
White	257 (73.4%)	29 (63.0%)	228 (75.0%)	26.1%
Comorbidities				
Current smoker	92 (26.3%)	20 (43.5%)	72 (23.7%)	42.9%
Diabetes	131 (37.4%)	18 (39.1%)	113 (37.2%)	4.0%
Hypertension	311 (88.9%)	41 (89.1%)	270 (88.8%)	1.0%
Congestive heart failure	50 (14.3%)	9 (19.6%)	41 (13.5%)	16.4%
Chronic kidney disease	53 (15.1%)	8 (17.4%)	45 (14.8%)	7.0%
Cancer	28 (8.0%)	3 (6.5%)	25 (8.2%)	6.5%
Sleep apnea	43 (12.3%)	4 (8.7%)	39 (12.8%)	13.4%
Prior Myocardial Infarction	91 (26.0%)	17 (37.0%)	74 (24.3%)	27.6%
BMI (Mean ± SD)	29.7 ± 6.0	29.4 ± 6.8	29.8 ± 5.8	6.1%
Prior Stroke/TIA	46 (13.1%)	6 (13.0%)	40 (13.2%)	0.3%
Baseline PHQ-8 (Mean ± SD)	4.7 ± 5.3	11.1 ± 6.5	3.7 ± 4.3	134.1%
Peripheral Artery Disease Severity and Treatment				
Invasive Treatment	121 (34.6%)	15 (32.6%)	106 (34.9%)	4.8%
ABI (Mean ± SD)	0.71 ± 0.19	0.73 ± 0.18	0.70 ± 0.20	15.7%
Socioeconomic Factors				
>high school education	307 (87.7%)	37 (80.4%)	270 (88.8%)	23.4%
Not enough months end finances	37 (10.7%)	11 (24.4%)	26 (8.6%)	43.6%
Avoid care due to cost	61 (17.6%)	14 (31.1%)	47 (15.6%)	37.3%
Medications at baseline				
Aspirin	307 (87.7%)	37 (80.4%)	270 (88.8%)	23.4%
Clopidogrel	136 (38.9%)	22 (47.8%)	114 (37.5%)	21.0%
Statin Post	277 (79.1%)	36 (78.3%)	241 (79.3%)	2.5%
SD= standard deviation, TIA=Transient Ischemic Attack, PHQ-8=8-Point Patient Health Questionairre, ABI=Ankle Brachial Index				

©2020 Malik AO et al. *JAMA Network Open*
